# Standardized guinea pig model for Q fever vaccine reactogenicity

**DOI:** 10.1371/journal.pone.0205882

**Published:** 2018-10-12

**Authors:** Laurie A. Baeten, Brendan K. Podell, Ann E. Sluder, Anja Garritsen, Richard A. Bowen, Mark C. Poznansky

**Affiliations:** 1 Colorado State University, Department of Biomedical Sciences, Fort Collins, Colorado, United States of America; 2 Colorado State University, Department of Microbiology, Immunology and Pathology, Fort Collins, Colorado, United States of America; 3 Massachusetts General Hospital, Vaccine and Immunotherapy Center, Boston, Massachusetts, United States of America; 4 InnatOss Laboratories BV, Oss, Netherlands; University of South Dakota, UNITED STATES

## Abstract

Historically, vaccination with *Coxiella burnetii* whole cell vaccines has induced hypersensitivity reactions in humans and animals that have had prior exposure to the pathogen as a result of infection or vaccination. Intradermal skin testing is routinely used to evaluate exposure in humans, and guinea pig hypersensitivity models have been developed to characterize the potential for reactogenicity in vaccine candidates. Here we describe a refinement of the guinea pig model using an alternate vaccine for positive controls. An initial comparative study used viable *C*. *burnetii* to compare the routes of sensitizing exposure of guinea pigs (intranasal vs intraperitoneal), evaluation of two time points for antigen challenge (21 and 42 days) and an assessment of two routes (intradermal and subcutaneous) of challenge using the ruminant vaccine Coxevac as the antigenic control. Animals sensitized by intraperitoneal exposure exhibited slightly larger gross reactions than did those sensitized by intranasal exposure, and reactions were more pronounced when skin challenge was performed at 42 days compared to 21 days post-sensitization. The intradermal route proved to be the optimal route of reactogenicity challenge. Histopathological changes at injection sites were similar to those previously reported and a scoring system was developed to compare reactions between groups receiving vaccine by intradermal versus subcutaneous routes. Based on the comparative study, a standardized protocol for assessment of vaccine reactogenicity in intranasally-sensitized animals was tested in a larger confirmatory study. Results suggest that screens utilizing a group size of n = 3 would achieve 90% power for detecting exposure-related reactogenic responses of the magnitude induced by Coxevac using either of two outcome measures.

## Introduction

Q fever is a zoonotic disease most often caused by exposure to infected ruminants and is prevalent in individuals with occupational exposures. Recent disease outbreaks in the Netherlands [1] and diagnosis among military personnel deployed to endemic regions [2–7] have highlighted this as a re-emerging infectious disease. The causative agent *Coxiella burnetii*, an obligate intracellular organism, is difficult to cultivate and diagnostic testing is inconsistent/unreliable in both humans and animals [8].

The four Q fever vaccines that have been developed for use in humans exhibit variable efficacy [9]. Furthermore, all four vaccines have been found to have significant side effects in vaccinees with prior sensitization to *C*. *burnetii*, variably resulting in fever, malaise and inoculation site granulomatous reactions [10–12]. These reactions are thought to be induced by a delayed hypersensitivity reaction to phase I LPS [12, 13]. As such there is a need for development of alternative effective vaccines that do not cause such hypersensitivity reactions in individuals previously exposed to *C*. *burnetii*.

In the evaluation of new vaccine candidates, a critical component is the ability to assess the potential for induction of hypersensitivity reactions. Guinea pig models have been described for the evaluation of vaccination reactions for the current vaccines in use [14–19]; the majority of these studies used inactivated bacterial suspensions to immunize guinea pigs prior to skin testing. Here we describe a refinement of the guinea pig model for evaluation of hypersensitivity reactions to *C*. *burnetii* antigens, undertaken during the course of an ongoing vaccine development project [20]. In a comparative study of selected protocol variables, we evaluated guinea pig sensitization by different routes of infection with live *C*. *burnetii*, tested two routes of antigen challenge, and assessed the time course of reactogenic responses following antigen challenge. We employed Coxevac, a whole cell vaccine approved for annual use in ruminants in Europe and reported to induce granulomatous reactions after repeated use in cattle [21], as a standardized antigen control to probe the responses of sensitized and unsensitized animals to different doses of vaccine antigen challenge. Based on the results of the comparative study, we defined a standardized protocol that was tested in a larger confirmation study to establish a framework for evaluation of the reactogenic potential of future Q fever vaccine candidates in the guinea pig model.

## Materials and methods

### Materials

#### Bacteria

*C*. *burnetii* Nine Mile strain (*Cb*9M) phase 1 RSA 411 (lot 3662550) was obtained from BEI Resources (Manassas, VA, USA). The phase 1 status of the stock prior to propagation was confirmed by the supplier by confirming reactivity with hyperimmune serum. An axenic bacterial working stock was prepared by a single expansion passage in acidified citrate cysteine medium (ACCM-2) pH 4.75 (Sunrise Science Products, San Diego, CA, USA). Propagation was as described [22], seeding cell free culture media with 1 x 10^6^ genome equivalents (ge) of the *Cb*9M BEI Resources stock. Flasks were incubated for nine days on a shaker (75 rpm) at 37˚C in a 2.5% O_2_, 5% CO_2_ environment. Bacteria were recovered by centrifugation at 14,000 x g for 30 min, resuspended in sucrose phosphate buffer [23] and stored at -80˚C. The working stock was found to contain 2.17 x 10^7^ ge per μL as determined by qPCR (see below). Infectivity of the working stock was confirmed in both mice (A/J) and guinea pigs. Reproducible induction of anti-*Coxiella* antibodies in guinea pigs following inoculation with the working stock at a standardized infective dose of 10^6^ ge [24] was confirmed prior to initiation of reactogenicity studies.

#### Vaccine

The vaccine, Coxevac, was obtained from CEVA Sante Animale (Lot 0101Eg1A, Libourne, France). This formalin inactivated phase I *C*. *burnetii* corpuscular antigen formulation is preserved with thiomersal and marketed for annual use in ruminants. Dosing in this study was based upon the assumption that Coxevac was standardized at 100 μg of antigen per mL [25]. The vaccine was determined to contain 86 μg of protein per mL (BCA protein assay, Pierce Biotechnology, Rockford, IL, USA) and qPCR indicated that the vaccine contained 1.3 x 10^8^ ge per mL.

### Experimental animals and ethics statement

#### Guinea Pigs

Female Dunkin-Hartley Crl:HA guinea pigs (300 g/35-42 days of age) were purchased from Charles River Laboratories, Wilmington, MA, USA. All guinea pigs were maintained under biosafety level 3 conditions in isolator cages (Smart Flow, Techniplast, Westchester, PA, USA) at the Regional Biocontainment Laboratory, Colorado State University, Fort Collins, Colorado, USA. Animals were provided water and guinea pig chow *ad libitum*. Dried fruit and hay pellets were offered for enrichment. Clinical evaluations were made daily on each animal throughout the study period to detect changes in body weight, body condition, behavior and activity level. Body temperature was documented at four-hour intervals by temperature recorders (Thermochron iButton, Maxim Integrated Products, Inc., Sunnyvale, CA, USA) implanted in the abdominal cavity via flank incision.

Animal research protocols were reviewed and approved by Colorado State University Institutional Animal Care and Use Committee (protocol number 16-6844A) and all activities were conducted in accordance with university and federal regulations.

### Methods

#### Quantitative PCR (qPCR)

Genomic DNA was extracted from samples using standard techniques (QIAamp DNA mini blood kit, Qiagen, Valencia, CA, USA) and qPCR was performed to detect *C*. *burnetii* targets using LSI VetMax *Coxiella burnetii* Absolute Quantification kit (Life Technologies, Lissieu, France).

#### Serology

Serological status was determined by ELISA using the Q Fever antibody test kit (IDEXX Laboratories Inc., Westbrook, ME, USA) with the secondary antibody replaced with peroxidase conjugated protein A/G at 1:10,000 dilution (Pierce Biotechnology, Rockford, IL, USA); the kit substrate is 3,3’,5,5’-tetramethylbenzidine (TMB). Results are presented as optical density at 450 nm (OD_450_). Antibody titers were also assessed by an indirect fluorescent antibody assay (IFA) using Q fever IFA substrate slides (Focus Diagnostics, Cyprus, CA, USA); staining methods as described by Kersh [26] were modified for use with guinea pig sera. Briefly, dilutions of sera (1:16 and 1:256) were added to wells on slides pre-coated with *C*. *burnetii* Phase I and Phase II and binding was assessed following reaction with DyLight 488 fluorochrome conjugated goat anti-guinea pig IgG (H+L) at 1:1,000 dilution (Thermo Fisher Scientific, Rockford, IL, USA). Titers ≥ 1:128 were considered positive for anti-*Coxiella* antibodies.

#### Guinea pig sensitization

Animals were chemically restrained (ketamine 40 mg/kg and xylazine 5 mg/kg, intraperitoneal (i.p.)) and inoculated with *Cb*9M by intranasal (i.n.) or i.p. administration with a target dose of 10^6^ ge [24] in a 100 μL volume of saline. Guinea pigs in the unsensitized control group received saline (100 μL) either i.p or i.n. Blood samples were collected from chemically restrained animals by venipuncture at days 0, 21, and 42 and at termination (day 28, 42, 49 or 63) for serum collection and evaluation of serological status.

#### Guinea pig antigen challenge and reactogenicity assessment

Hypersensitivity testing was modified from that described by Wilhelmsen and Waag [14]. At day 21 or 42 post-infection, guinea pigs were chemically restrained (ketamine 40 mg/kg and xylazine 5 mg/kg, i.p.) and the dorsal lumbar surface was shaved. Subcutaneous (s.c.) and intradermal (i.d.) inoculations were made in 100 μL volumes with saline or vaccine at 1, 2, or 4 μg. The inoculation sites were evaluated at 8 hours post-injection and daily thereafter for 7 or 21 days. The diameter of erythema and thickness of induration at each inoculation site were measured with calipers. At 7 or 21 days post-challenge, inoculation sites were excised from euthanized animals by punch biopsy (12mm AcuPunch, Acuderm Inc., Ft. Lauderdale, FL, USA) and organs (heart, lung, liver, spleen) were collected in 10% formalin for paraffin embedding and sectioning. Sections were stained with hematoxylin and eosin for histopathological analysis. The extent of histological lesions in each of two non-adjacent skin biopsy sections from i.d. and s.c. inoculation sites were evaluated by a single blinded pathologist (BKP) in their entirety to develop a scoring system on a graded scale of 0–5 (Table 1). The entire slide set was then re-evaluated in a blinded manner to obtain final scores. All sections were deemed appropriate for scoring as all contained targeted areas of dermis and subcutaneous tissue.

**Table 1:** Histopathological scoring criteria (0–5) for guinea pig inoculation sites.

Score	Histopathological Description of Skin Biopsy Sites
**0**	No inflammatory changes present, tissue is within normal limits
**1**	1–3 distinct and small aggregates of macrophage dominated inflammation. Other cell types are rare. Inflammation is localized to the deep dermis without tissue infiltration.
**2**	More extensive macrophage dominated inflammation with coalescing foci or linear infiltrate in the deep dermis. Neutrophils are present. There is limited tissue infiltration and no tissue destruction or edema.
**3**	Macrophage dominated inflammation remains the principal component. Lymphocytic inflammation is present to more extensive degree. Neutrophils are not associated with tissue necrosis. Inflammatory infiltrate extends into the deep dermis and superficial subcutis and along tissue planes to both endos of the biopsy specimen. Edema is uncommon.
**4**	Inflammation is increasingly pyogranulomatous. Lymphocytes are less common and often peripheral to granulomatous inflammation. Edema is present and inflammation extends into the superficial dermis ad deep into the subcutis. Necrosis is absent.
**5**	Similar to a score of 4, the inflammation is widespread and pyogranulomatous; however, these most severely affected samples have one to multiple foci of liquefactive necrosis with or without collagen degeneration and a very high proportion of neutrophils.

#### Statistics

Statistical analyses were performed using GraphPad PRISM (version 5). Group mean comparisons using one-way ANOVA with Tukey’s multiple comparison test or unpaired t test (one-tailed) were used to evaluate differences among groups. Unless otherwise noted P values of ≤0.05 were considered statistically significant.

Estimates of statistical power and group size requirements were made using computational tools available from the MGH Biostatics Center (http://hedwig.mgh.harvard.edu/sample_size/size.html) and the University of Iowa Department of Statistics and Actuarial Science (Lenth, R.V., Java Applets for Power and Sample Size, Retrieved 21 September 2018; http://www.stat.uiowa.edu/~rlenth/Power).

## Results

### Comparative study of bacterial and antigen exposure routes

**Fig 1:**
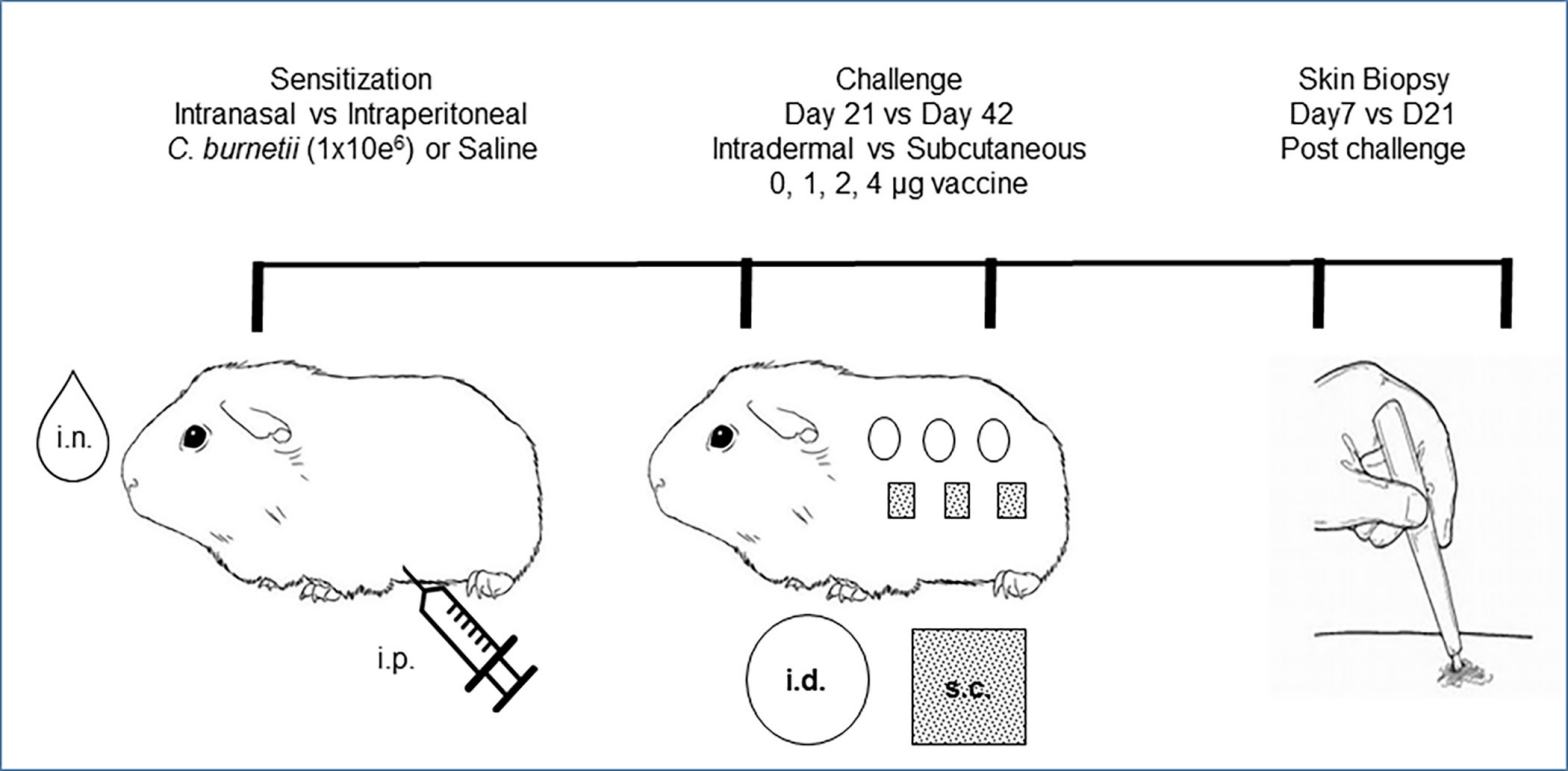
Guinea pig reactogenicity model.

A comparative study was designed to evaluate reactogenic responses in the guinea pig model using different routes of sensitizing exposure to *C*. *burnetii* and of antigen challenge. Guinea pigs (n = 24) were randomly assigned to three groups (Fig 1). Experimental animals were inoculated with a standardized dose of a defined working stock of *Cb*9M by either intranasal (i.n.) or intraperitoneal (i.p.) administration (n = 8 per group). Guinea pigs in the unsensitized control group (n = 8) received saline either i.p or i.n. (n = 4 each route). At day 21 or 42 post-inoculation (p.i.), antigen challenge inoculations were made subcutaneously (s.c.) or intradermally (i.d.) as described in the Methods (n = 4 at each challenge time point). Each animal received inoculations of saline and Coxevac vaccine at 1, 2, and 4 μg at distinct challenge sites on the back. Two animals per challenge time point were terminated and tissues were harvested for histopathology at 7 days and at 21 days post-challenge (p.c.) (days 28 and 42 p.i. for animals challenged at day 21 p.i.; days 49 and 63 p.i. for animals challenged at day 42 p.i.). Reactions to antigen challenge were assessed by daily inspection for erythema and induration and by histopathology of challenge site skin biopsies taken at termination, as described in the Methods.

### Clinical responses to inoculation with Cb9M

**Fig 2:**
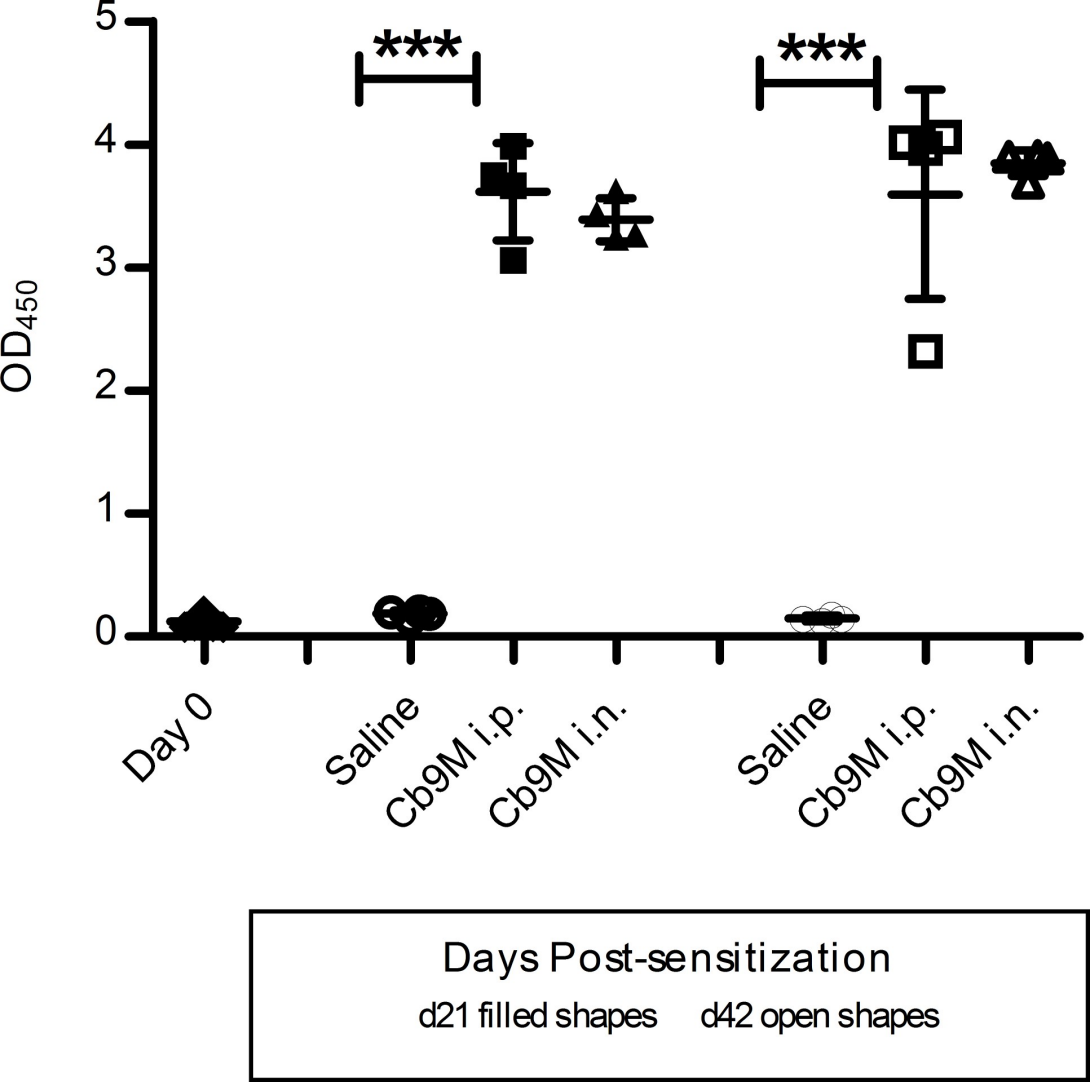
Q fever antibody ELISA results. Data are represented as group mean optical density values (OD_450_) determined using the IDEXX Q fever antibody ELISA. Anti-*Coxiella* antibody measured in a 1:1,000 dilution of serum collected from guinea pigs at day 0, day 21 (filled shapes) and 42 (open shapes) post-inoculation with *C*. *burnetii* Nine Mile. Day 0 antibody levels were similar to saline controls. Data are presented by route of inoculation (intraperitoneal i.p.; intranasal i.n.; saline controls). Error bars represent standard deviations. Group mean OD_450_ were statistically different from saline controls (one way ANOVA with Tukey’s multiple comparison test; ***P = <0.0001).

Anti-*C*. *burnetii* immune responses were confirmed in all guinea pigs inoculated with *Cb*9M by detection of antibody prior to vaccine antigen challenge (day 21 or 42 p.i.). No anti-*C*. *burnetii* antibody was detected by ELISA in guinea pig sera at day 0 (mean OD_450_ 0.118 ± 0.034) or in saline-inoculated guinea pigs at the time of antigen challenge (day 21 or 42 p.i.; group mean OD_450_ 0.156 ± 0.046). In contrast, high levels of antibody were detected in serum collected from *Cb*9M-inoculated animals. At day 21 p.i. serum antibody levels were characterized by mean OD_450_ values of 3.616 ± 0.198 for the i.p. group and 3.391 ± 0.087 for the i.n. group. At day 42 p.i., mean OD_450_ values were 3.593 ± 0.425 for the i.p. group and 3.847 ± 0.052 for the i.n. group. No significant differences in OD_450_ values (p = 0.625) were noted between inoculation routes or days post-challenge (Fig 2), although OD_450_ values near the maximum detection limit for the instrumentation used for this assay (4.600) limited discrimination between robustly responding groups. Antibody titers, as measured by IFA, were found to be ≥ 1:256 in all *C*. *burnetii-*inoculated guinea pigs.

**Fig 3:**
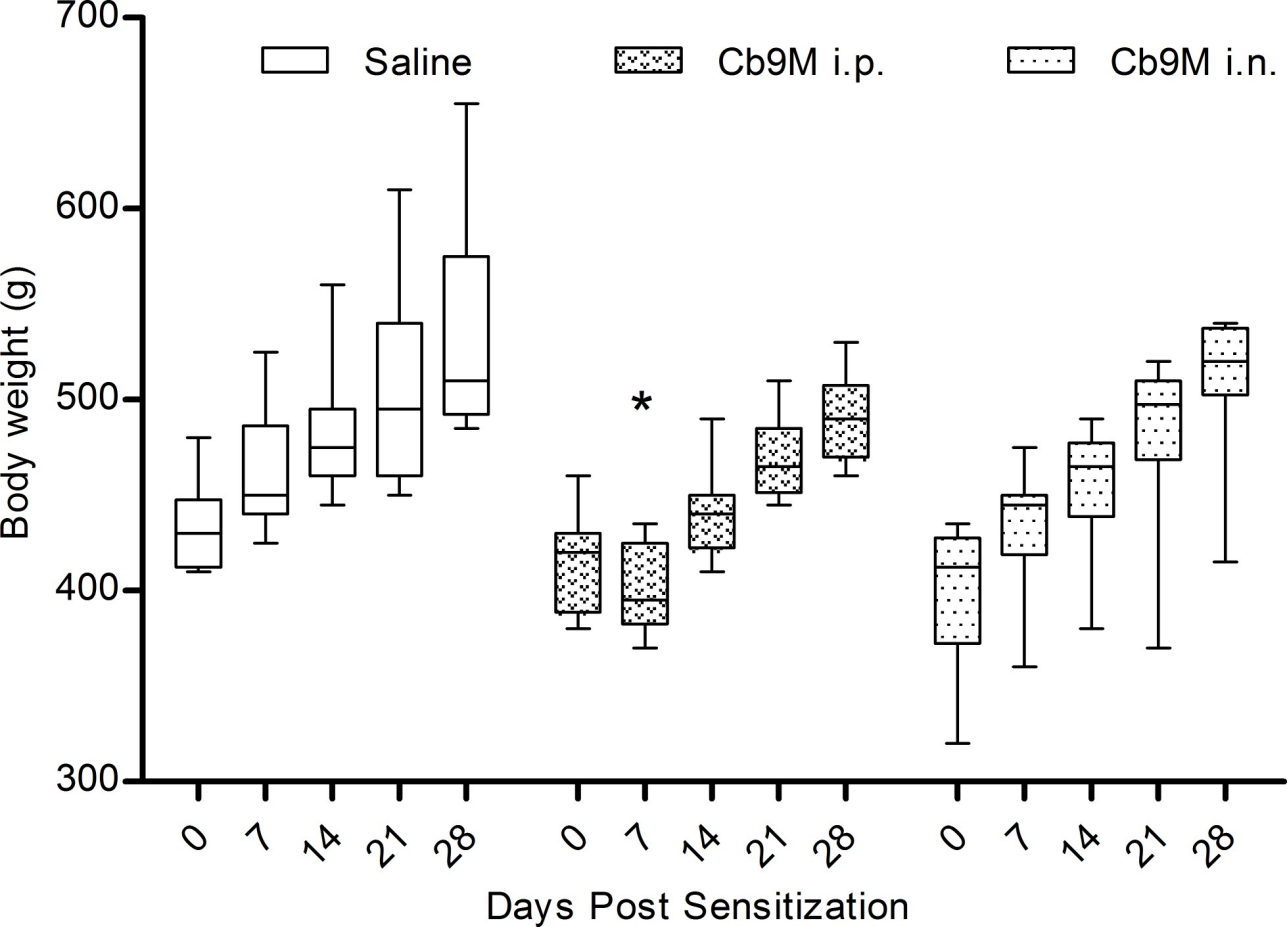
Weekly body weights by inoculation route. Data are represented as group mean (n = 8) by route of exposure to *C*. *burnetii* Nine Mile (*Cb*9M, i.p. intraperitoneal; i.n. intranasal; saline controls). Error bars represent standard deviation; boxes denote 25^th^ and 75^th^ quartiles. Group mean body weights of saline and i.p. inoculated groups were statistically different at day 7 p.i. (one-way ANOVA with Tukey’s multiple comparison test; *P = 0.003).

Despite the antibody responses indicating bacterial exposure, no apparent changes in guinea pig behavior or disease symptoms were noted following inoculation with *Cb*9M. The group mean body weight at day 0 was 416.5 gm (range 320–480 g). Guinea pigs inoculated by the i.p. route experienced a 4% drop in weight (mean 28 g; range 5–60 g) between days 0–7 p.i., which was statistically different from weights of saline-inoculated control guinea pigs in the same time window (p value = 0.003) (Fig 3). During this time the i.n. inoculated group did not experience weight loss, and though they exhibited less weight gain than did saline controls the differences were not statistically significant. Body temperature, as determined by i.p. iButton readings, generally remained within the normal range throughout the study; the group baseline mean body temperature was 39.2˚C compared to the published normal range of 38–40˚C [27]. Transient body temperature spikes were noted between days 4 and 8 in the i.p. inoculated group (mean 40.2˚C; range 38.9–41.6˚C). Mean group body temperatures remained within the normal range for the i.n. inoculation group, with two of four individuals experiencing a transient spike (< 4 hours) up to 41.5˚C between days seven and ten. Body temperatures remained within the normal range for all animals following antigen challenge.

### Intradermal and subcutaneous challenge reactions

**Fig 4:**
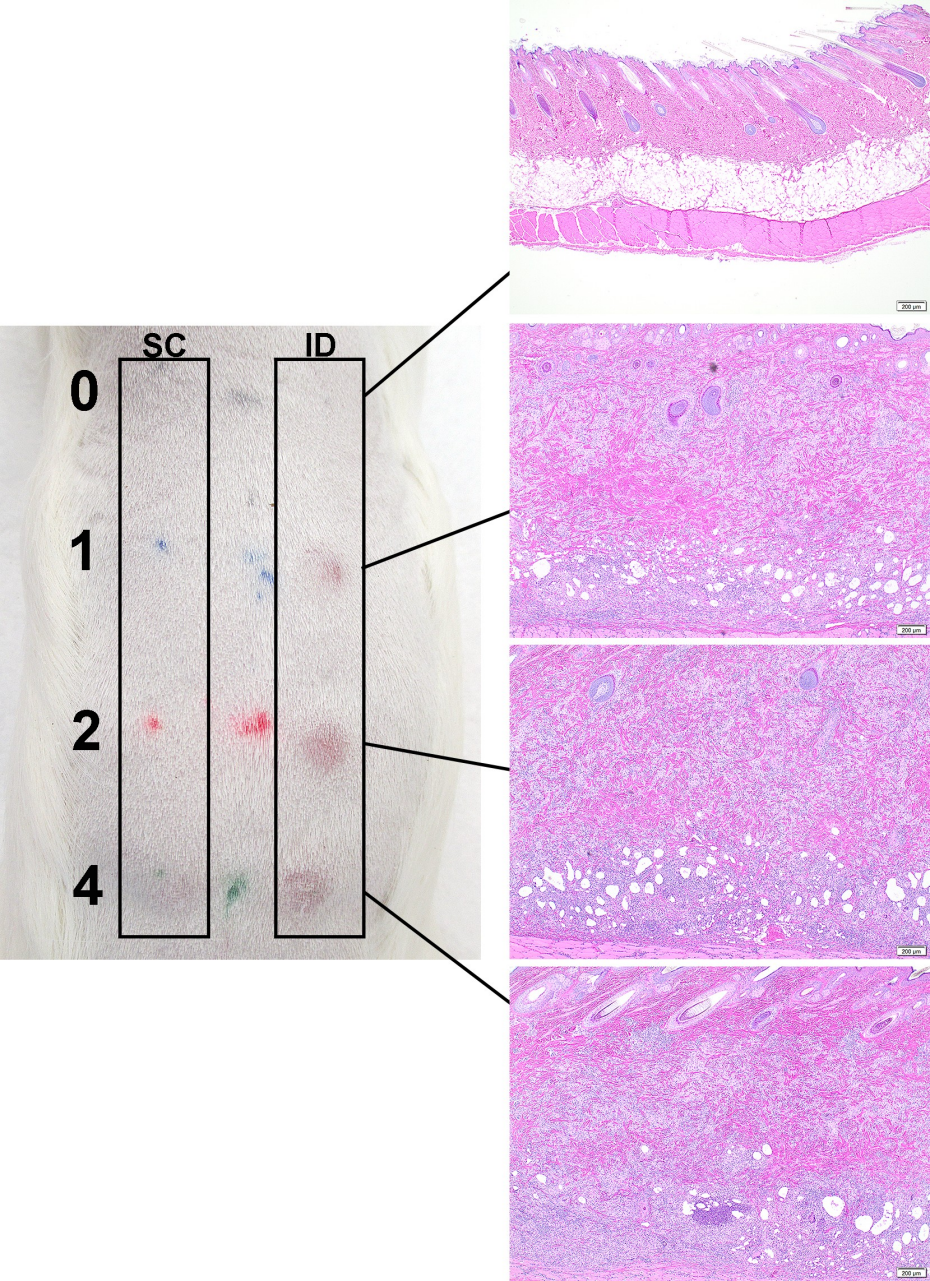
Skin lesions at day 21 post challenge. The left panel depicts the gross lesions observed at day 21 post challenge in a guinea pig sensitized by intranasal inoculation (challenge at day 42 post-inoculation). Vaccine challenge dose is indicated by the numbers on the far left (0, 1, 2, 4 μg). SC: subcutaneous inoculation sites are on the left, ID: intradermal inoculation sites on the right. Corresponding photomicrographs in the right panel demonstrate the dose-dependent increase in lesion severity, evident particularly among the intradermal inoculation sites, compared to the absence of any inflammation in saline inoculations (0 μg).

**Fig 5:**
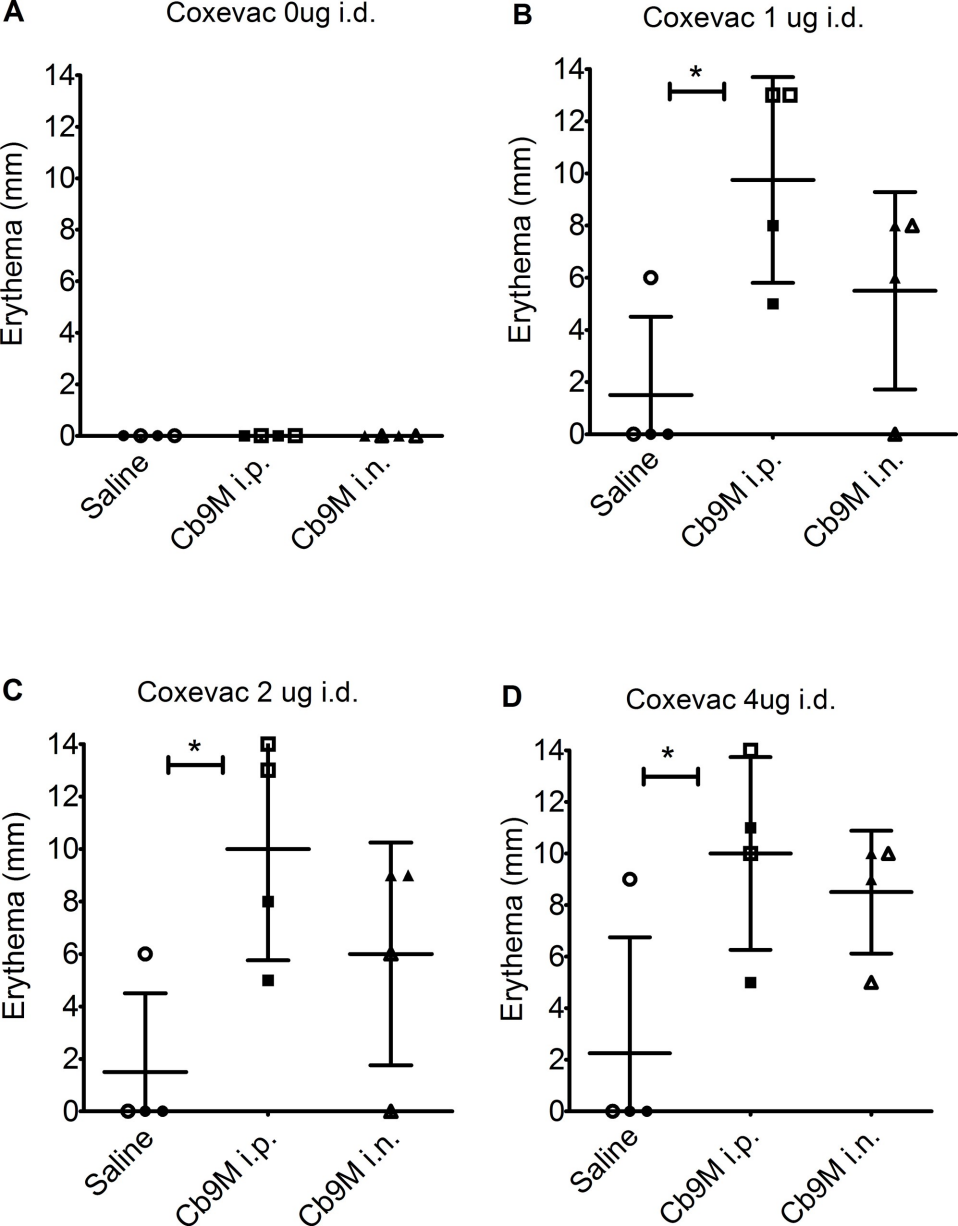
Zone of erythema at D42 intradermal challenge sites. Data are shown as the zone of erythema (mm) at intradermal vaccine injection sites challenged at day 42 p.i., grouped by route of sensitization with *C*. *burnetii* Nine Mile (*Cb*9M) (i.p., intraperitoneal; i.n., intranasal) or saline controls. Measurements are presented for terminally harvested injection sites at day 7 p.c. (open shapes) and day 21 p.c. (filled shapes). Values are presented by challenge dose (A) 0 μg Coxevac, (B) 1 μg Coxevac, (C) 2 μg Coxevac, and (D) 4 μg Coxevac. ^1^Histopathological scoring (0–5) is as noted in Table 1. Error bars represent group mean and standard deviations. Group mean differences assessed by one-way ANOVA with Tukey’s multiple correction test; *P≤0.05.

Sensitization of *Cb*9M-inoculated animals to *Coxiella* antigens was confirmed using the formalin-inactivated phase I whole cell vaccine Coxevac as a standardized challenge antigen. Responses to both s.c. and i.d. antigen challenge were assessed. Gross abnormalities were not observed at the s.c. inoculation sites in animals until day 10 p.c., with some reactions persisting until day 21 p.c. These reactions appeared as minor subcutis swelling with no erythema or induration noted. The i.d. vaccine challenge sites in *Cb*9M-inoculated animals developed detectable erythema and induration at all doses of vaccine tested (Fig 4), generally within eight hours after inoculation. Reactions were most consistent at 2 and 4 μg doses of vaccine, and most severe (visibly darker) at day 42 p.i. challenge inoculation sites relative to the day 21 p.i. challenge sites (Table 2). In general, i.d. reaction size in *Cb*9M-inoculated animals reached full extent by three to seven days p.c. and persisted until at least day 21 p.c. (the longest time point assessed; Figs 4 and 5; S1 Fig). Gross reactions at i.d. challenge sites were also noted in some saline control animals, though visible erythema generally decreased in these animals by day 21 p.c. (S1 Fig). No reactions were observed to challenge with a control saline solution in either saline- or *C*. *burnetii*-sensitized animals.

**Table 2:** Zone of erythema at intradermal challenge sites.

Sensitization Route^1^	Saline				Intraperitoneal				Intranasal			
	Coxevac (μg)				Coxevac (μg)				Coxevac (μg)			
	0	1	2	4	0	1	2	4	0	1	2	4
Day 21 PS^2^												
7 d PC^3^	0	0	0	0	0	0	0	10.5	0	0	3.5	4.5
21 d PC	0	0	5.5	5.8	0	3.3	3.3	4.5	0	3.0	7.5	6.0
Day 42 PS^2^												
7 d PC^3^	0	3.3	3.0	4.5	0	12.3	12.8	11.3	0	3.5	3.0	7.8
21 d PC	0	0	0	0	0	6.5	6.3	10.0	0	6.3	8.3	8.5

Data are listed as the zone of erythema (mm) at intradermal sites by sensitization route, day of challenge and dose of vaccine. Values are represented as group mean (n = 2).

^1^Guinea pigs were sensitized to *C*. *burnetii* Nine Mile strain by intraperitoneal or intranasal inoculation. Unsensitized guinea pigs were inoculated with saline.

^2^Guinea pigs were challenged with vaccine (Coxevac) at 0, 1, 2, 4 μg by intradermal inoculation at days 21 or 42 post-sensitization (PS).

^3^The mean diameter (mm) of zones of erythema at intradermal inoculation sites were measured after re-shaving of the inoculation sites at days 7 or 21 post-challenge (PC).

### Histological changes

**Fig 6:**
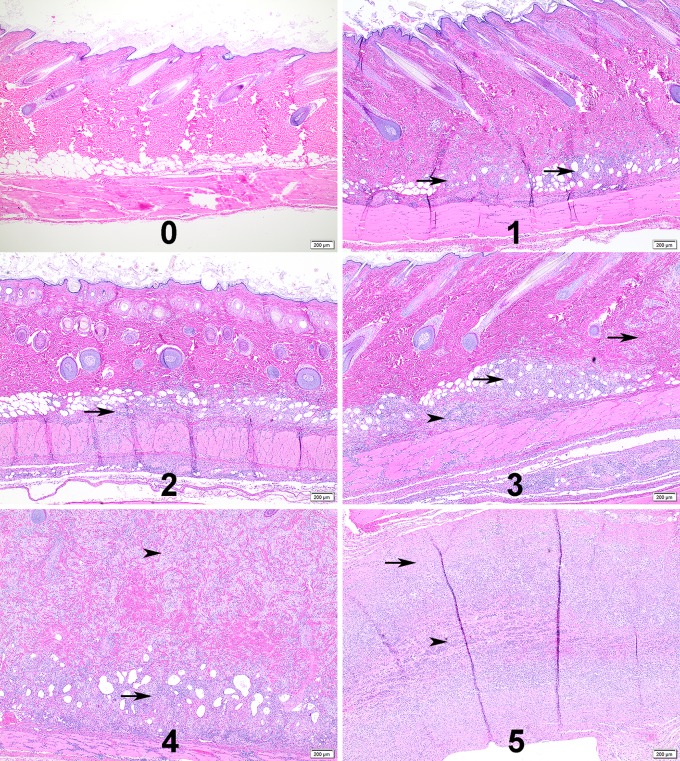
Representative histopathological scoring. Representative photomicrographs of guinea pig skin inoculation sites for the histological scoring criteria used in the study. Numbers represent the histological scores of 0–5. 1) Arrows, multiple small aggregates of macrophage dominated inflammation in the deep dermis. 2) Arrow, linear infiltrate in deep dermis with extension into the panniculus muscle. 3) Arrows, more extensive macrophage dominated inflammation with extension through the dermis; Arrowhead, increased frequency of lymphocytic inflammation. 4) Arrow, deep dermis is effaced by dense inflammation; Arrowhead, majority of the dermis is replaced by macrophage dominated inflammation. 5) Arrow, skin is replaced by pyogranulomatous inflammation; Arrowhead, central area of necrosis contains an abundance of neutrophils.

**Fig 7:**
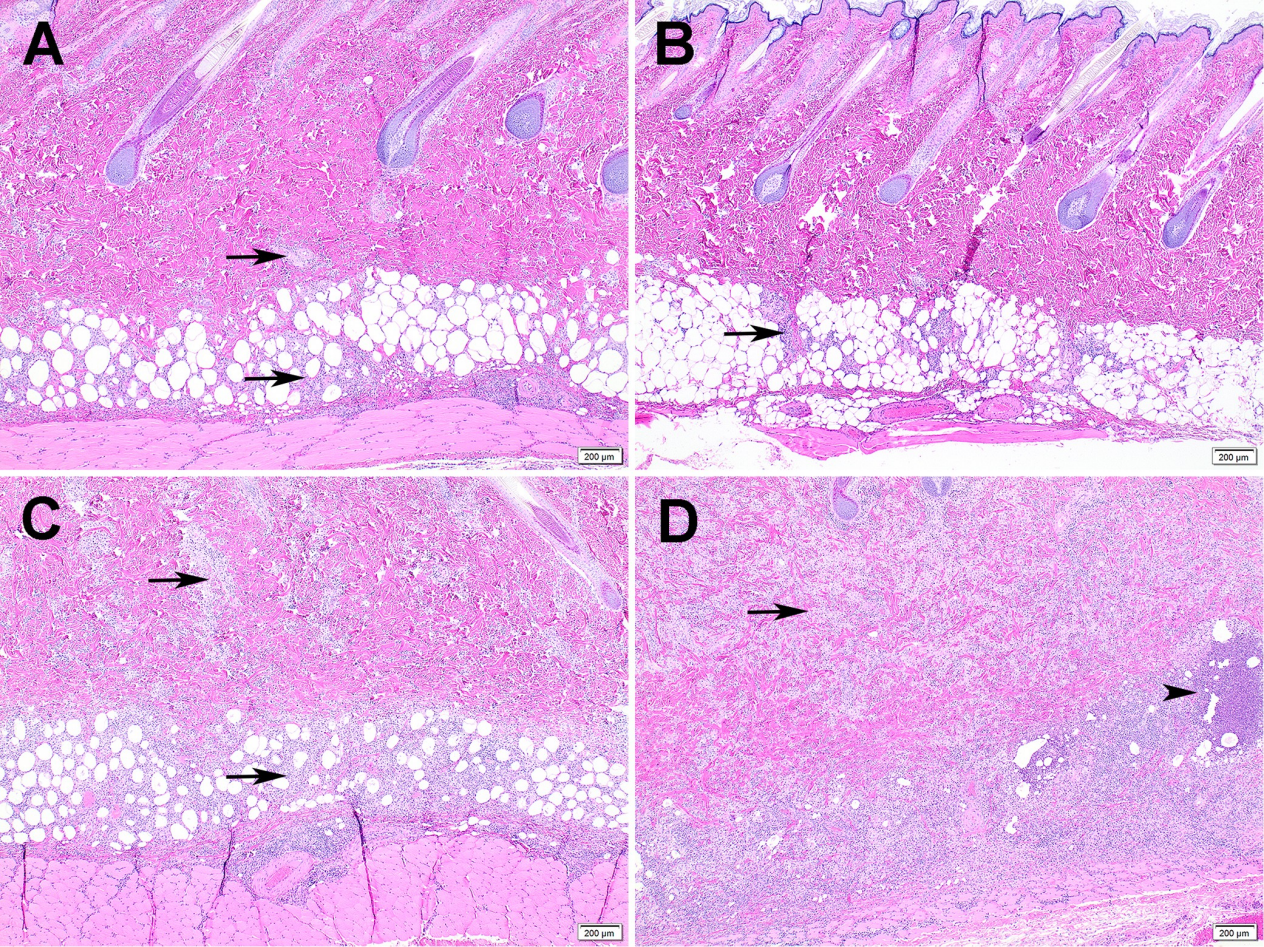
Reactogenicity to vaccine in sensitized vs unsensitized guinea pigs. Images are representative of guinea pigs inoculated i.n. with *C*. *burnetii* (C and D) or unsensitized (saline) (A and B), then challenged with a 2 μg intradermal dose of vaccine (Coxevac) at day 42 p.i.. A) Inflammation at day 7 p.c. in unsensitized guinea pig is multifocal (arrows) and limited to the deep dermis. B) Inflammation at day 21 p.c. in unsensitized guinea pig is similar to day 7 (arrow). C) More severe inflammation with extension through the dermis (arrows) at day 7 p.c. in *Cb*9M-sensitized guinea pig. D) Progression of inflammation in *Cb*9M-sensitized guinea pig at day 21 p.c. (arrows) with increased frequency of neutrophils and presence of microabscesses (arrowheads).

**Fig 8:**
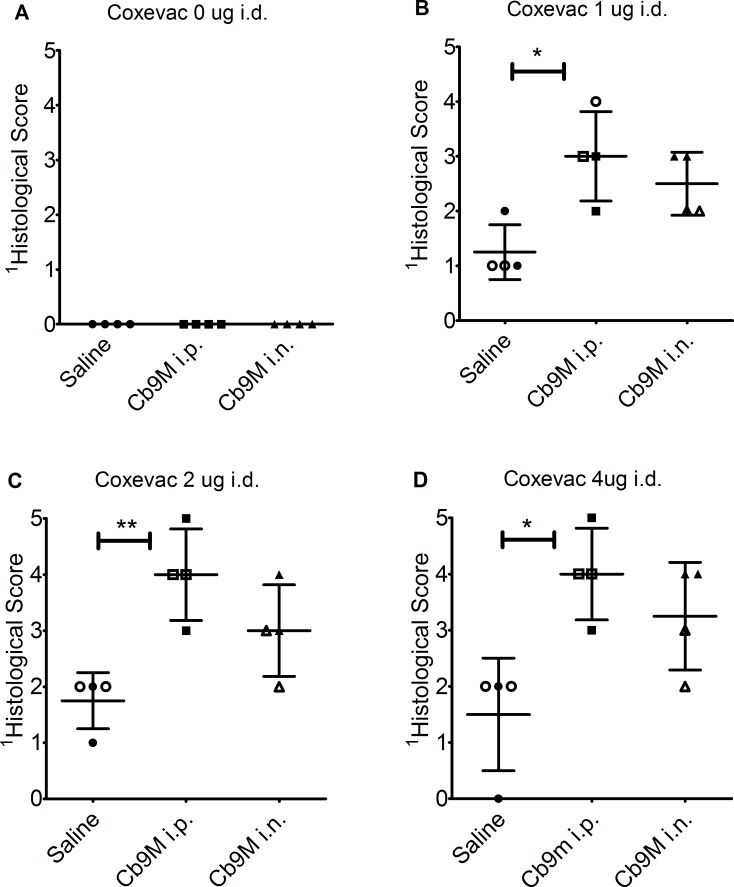
Histological scores at D42 intradermal challenge sites. Data are presented as histological scores at termination, following day 42 p.i. intradermal challenge, grouped by route of sensitization with *C*. *burnetii* Nine Mile (*Cb*9M) (i.p., intraperitoneal; i.n., intranasal) or saline controls. Scores are shown for injection sites harvested at day 7 p.c. (open shapes) and day 21 p.c. (filled shapes). Values are presented by challenge dose (A) 0 μg Coxevac, (B) 1 μg Coxevac, (C) 2 μg Coxevac, and (D) 4 μg Coxevac. ^1^Histopathological scoring (0–5) is as noted in Table 1. Error bars represent group mean and standard deviations. Group mean differences assessed by one-way ANOVA with Tukey’s multiple correction test; *P≤0.05; ** P≤0.01.

Visceral organ lesions in sensitized animals were limited to a few microgranulomas in the liver and sporadic hepatocellular necrosis. Splenic lesions were limited to increased neutrophils without tissue destruction. No significant differences in organ lesions were noted between animals sensitized using the i.p. and i.n. inoculation routes. Histopathological evaluation of guinea pig skin reactions at vaccine inoculation sites utilized scoring criteria based upon the cellular composition, distribution of inflammation, overall size of the inflammatory response, infiltration of surrounding tissue, vascular sequelae and presence/absence of tissue injury and/or necrosis (Table 1 and Fig 6). Inflammation was observed at both intradermal and subcutaneous vaccine inoculation sites in both unsensitized and *Cb*9M-sensitized guinea pigs (Fig 7). Inflammation was more severe in *Cb*9M-sensitized animals, and at intradermal challenge sites compared to subcutaneous challenge sites, consistent with the observed levels of gross erythema and induration. The degree of inoculation site inflammation was proportional to vaccine dose, with very little difference noted between 2 and 4 μg doses (Table 3 and Fig 8). The inflammation was more severe overall in guinea pigs sensitized by the i.p. route compared to the i.n. route. In sensitized animals microabscesses were more frequently observed at day 21 p.c. than at day 7 p.c. (Fig 7), consistent with ongoing inflammatory tissue damage. Among the unsensitized guinea pigs, there was limited histopathological difference between skin samples collected at day 7 p.c. and day 21 p.c. indicating that vaccine alone can induce a granulomatous inflammation that fails to resolve within the time frame evaluated but does not result in progressive tissue damage.

**Table 3:** Histopathological scores by vaccine challenge dose.

	Histological Scores^1^			
i.d. Challenge^2^	0 μg	1 μg	2 μg	4 μg
**Day 21 PS**^3^				
Saline 7 d PC^4^	0	1	0.5	1.5
Saline 21 d PC	0	0.5	1.5	2
*Cb*9M i.p. 7 d PC	0	2	2.5	2.5
*Cb*9M i.p. 21 d PC	0	3	3.5	3.5
*Cb*9M i.n. 7 d PC	0	3	3	3
*Cb*9M i.n. 21 d PC	0	2	3	3.5
**Day 42 PS**^3^				
Saline 7 d PC	0	1	2	2
Saline 21 d PC	0	1.5	1.5	1
*Cb*9M i.p. 7 d PC	0	3.5	4	4
*Cb*9M i.p. 21 d PC	0	2.5	4	4
*Cb*9M i.n. 7 d PC	0	2	2.5	2.5
*Cb*9M i.n. 21 d PC	0	3	3.5	4

Data are presented as group mean scores (n = 2) for each sensitization inoculation (saline, *Cb*9M: *Coxiella burnetii* Nine Mile, i.p.: intraperitoneal, i.n.: intranasal), challenge dose and post-challenge time point (day 7, day 21).

^1^Histopathological scoring as noted in Table 1.

^2^Intradermal (i.d.) vaccine (Coxevac) challenge dose (0, 1, 2 and 4 μg).

^3^Guinea pigs were challenged at day 21 or day 42 post-sensitization (PS) with *Cb*9M.

^4^The mean histological scores of challenge sites at day 7 or 21 post challenge (PC).

### Reproducibility of standardized protocol using i.n.-sensitization

The results of the initial comparative study indicated that detectable sensitization to *Coxiella* antigens was achieved using both i.p. and i.n. infection routes, with i.p.-sensitized animals showing more pronounced reactions compared to saline control animals (Fig 5 and Fig 8). The i.n. route is a better mimic of the natural route of exposure leading to antigen sensitization and vaccine reactogenicity in humans. Therefore, an expanded replicate study was performed to confirm the reproducibility of guinea pig sensitization following i.n. exposure, and to estimate the group size that would be required to detect a hypothesized difference in reaction severity between control and i.n.-sensitized animals. Preliminary estimates made using the limited data available from the comparative study suggested that a group size of 4–12 animals would have 80% power to detect a difference between control and i.n.-sensitized animals, depending on the effect size hypothesized. Based on these estimates and the capacity of the BL3 facility for housing an extended guinea pig study, the replicate study utilized group sizes of n = 9 for *Cb*9M-sensitized animals and n = 4 for saline control animals.

**Fig 9:**
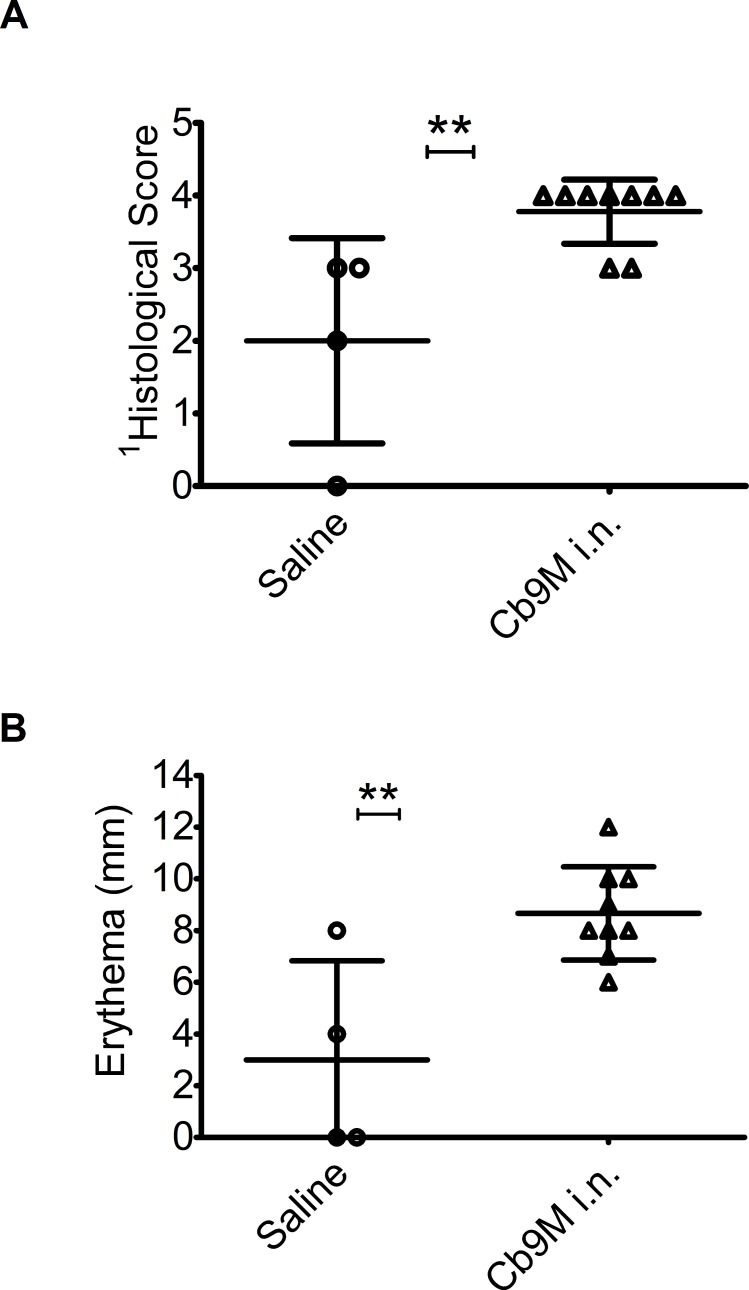
Zone of erythema and histopathological scores after antigenic challenge of i.n. sensitized guinea pigs. Data are presented for each inoculation group (saline (n = 4), *Cb*9M: *C*. *burnetii* Nine Mile, i.n.: intranasal, (n = 9)). Coxevac challenge (2 μg delivered i.d.) was performed at day 42 p.i. (A) Histopathological scoring (0–5) is as noted in Table 1, (B) zone of erythema (mm). Error bars represent group mean and standard deviations. **Group means were statistically different for both histological scores and erythema (P value 0.002, 0.002 respectively, assessed by one-sided t test).

Antigenic challenge was standardized as 2 μg Coxevac administered i.d. 42 days after infection. Reactions were assessed 7 days post-challenge, when erythema was expected to have reached full extent but before milder reactions may have begun to resolve. As in the initial study, reactions to antigenic challenge were observed in all *Cb*9M-sensitized and some saline control guinea pigs (Fig 9). No reactions were observed in response to negative control saline challenge. Reactions were more severe and consistent in the *Cb*9M-sensitized animals than in the control animals, as measured both by extent of erythema (P = 0.002) and by histopathology (P = 0.002). Based on these data, group sizes of n = 3 would have ≥90% power to detect a difference in mean reaction severity between control and sensitized animals of the magnitude observed in this study, at a significance level of 0.05 in a one-sided t test, using either erythema or histological score as an outcome measure.

## Discussion

Guinea pigs have been used for *Coxiella* infection models since the identification of this pathogen in the early 1950s. Guinea pigs are also used as the animal model of choice for the evaluation of several other hypersensitivity reactions such as contact dermatitis [28], responses to respiratory allergens and irritants [29], drug reactions [30] and reaction to other infectious disease agents [31, 32].

The term reactogenicity was coined by the Federal Drug Administration to describe the expected adverse vs unexpected (local and systemic) reactions noted during clinical trials of drugs, vaccines, and other pharmaceuticals [33]. In most cases, these reactions were secondary to adjuvants and/or were short term reactions with no long term sequelae. Reactions were scored 0–4 with regard to fever, injection site pain, induration and erythema [34]. In the case of *C*. *burnetii* vaccines, granulomatous reactions are common and can persist for several weeks [35, 36].

The reactogenicity model described here using viable organism for sensitization and a ruminant vaccine as an antigenic control exhibited similar reactions to those reported by others [14, 15]. Our findings confirm reactogenicity is not strain dependent as skin reactions to Coxevac (*Cb*9M) were similar to those reported using antigen preparation from the Henzerling and Ohio strains [16] and the California 76 strain [18]. In addition, the skin lesions observed in guinea pigs indicate that Coxevac gives comparable reactions to those reported for Q-Vax [14, 15], the vaccine currently available for use in humans (Seqirus, Victoria, Australia).

Reactogenic responses of *Cb*9M-sensitized guinea pigs to antigen challenge were relatively stable overall to changes in several protocol variables, using two measures of response (visible extent of erythema and histopathological scoring). Our study provides additional information with respect to routes of sensitization with viable organism and challenge with Coxevac. In the context of model standardization, the i.p. route is likely to deliver a more consistent dose of organism compared to the i.n. route. However, the i.n. route is a better mimic of the natural route of exposure, and we demonstrated that a standardized i.n. dose of viable *C*. *burnetii* reproducibly sensitized animals to subsequent vaccine antigen challenge.

The i.d. challenge route is preferable to the s.c. route in a standardized screening model as the gross reactions are more easily detected and more consistent following i.d. challenge. The i.d. route also mimics the pre-vaccination screening currently used in humans [15, 37]. In our initial comparative study, consistent reactions were observed when the Coxevac antigen challenge was administered either 21 or 42 days after the sensitizing infection, suggesting that there is an operational window within which a reactogenicity screening protocol can be standardized to accommodate the work flow of a given program. In our confirmatory study we administered antigen challenge at day 42 p.i., as reactions were generally more pronounced at this challenge time in the comparative study.

A range of Coxevac vaccine doses elicited detectable reactions in *Cb*9M-sensitized guinea pigs, with visible erythema peaking by 3–7 days post-challenge and persisting throughout a 21-day post-challenge monitoring period. More variable reactions were observed in some unsensitized control guinea pigs, with any visible erythema generally diminishing by 7–10 days post-challenge. Histological evaluation of challenge site biopsies revealed generally mild inflammation in some control animals, with little difference between biopsies taken at day 7 and day 21 post-challenge. In contrast, challenge site biopsies from *Cb*9M-sensitized animals exhibited pronounced inflammation at day 7 p.c., with more extensive inflammation and the presence of microabscesses observed at day 21 p.c., consistent with ongoing inflammatory tissue damage. These results suggest that when testing novel vaccine candidates, assessment at day 7 p.c. should allow detection of acute reactions prior to resolution of milder reactions, while examination at longer post-challenge time points can identify sustained and progressive reactions.

The standardized protocol utilized in our confirmatory study is designed to be used in initial evaluation of the reactogenicity potential of novel Q fever vaccine candidates or vaccine components. The results of the confirmatory study suggest that screens utilizing group sizes of n = 2 or n = 3 would achieve 80% or 90% power, respectively, to detect exposure-related reactogenic responses of the magnitude induced in intranasally-sensitized guinea pigs by i.d. antigen challenge with 2 μg Coxevac. Milder reactions observed in an initial assessment of a novel vaccine would need to be further confirmed with a larger follow up study. Characterization of any observed reactogenic effects in the guinea pig model can inform preclinical and clinical vaccine development decisions. For example, if a novel vaccine elicits comparable reactions in *Coxiella*-sensitized and control animals, an assessment of lower vaccine doses in control and sensitized animals can be performed to determine if the vaccine is differentially reactogenic or a general irritant. If a vaccine candidate induces a reaction by day 7 p.c. in the standardized protocol described here, further evaluation would be of interest to determine if the reaction resolves more rapidly than does the reaction to Coxevac, since an efficacious vaccine with shorter-lived side effects than those observed with whole cell vaccines could still have value.

## Supporting information

S1 Fig Daily zone of erythema at intradermal challenge sites after day 42 challenge. Data are shown as the zone of erythema (mm) at intradermal vaccine injection sites after day 42 p.i. challenge, grouped by challenge dose and route of sensitization with C. burnetii Nine Mile (Cb9M) (i.p., intraperitoneal; i.n., intranasal) or saline controls. Measurements are presented as group mean (n = 2) from day 0 to day 21 post-challenge.
(TIF)

## References

[1] Roest HIJ, Tilburg JJHC, van der Hoek W, Vellema P, van Zijderveld FG, Klaassen CHW, et al The Q fever epidemic in The Netherlands: history, onset, response and reflection. Epidemiology and Infection. 2011;139(1):1–12. 10.1017/S0950268810002268 20920383

[2] Anderson AD, Baker TR, Littrell AC, Mott RL, Niebuhr DW, Smoak BL. Seroepidemiologic Survey for Coxiella burnetii Among Hospitalized US Troops Deployed to Iraq*. Zoonoses and Public Health. 2011;58(4):276–83. 10.1111/j.1863-2378.2010.01347.x. ISI:000290486000008. 20880090

[3] Faix DJ, Harrison DJ, Riddle MS, Vaughn AF, Yingst SL, Earhart K, et al Outbreak of Q fever among US military in western Iraq, June-July 2005. Clin Infect Dis. 2008;46(7):e65–8. Epub 2008/05/01. 10.1086/528866 . 18444807

[4] Leung-Shea C, Danaher PJ. Q fever in members of the United States armed forces returning from Iraq. Clin Infect Dis. 2006;43(8):e77–82. 10.1086/507639 . 16983603

[5] Anderson AD, Smoak B, Shuping E, Ockenhouse C, Petruccelli B. Q fever and the US military. Emerg Infect Dis. 2005;11(8):1320–2. 10.3201/eid1108.050314 ; PubMed Central PMCID: PMCPMC3320491. 16110586 PMC3320491

[6] Gleeson TD, Decker CF, Johnson MD, Hartzell JD, Mascola JR. Q fever in US military returning from Iraq. Am J Med. 2007;120(9):e11–2. 10.1016/j.amjmed.2007.03.020 . 17765028

[7] Hartzell JD, Peng SW, Wood-Morris RN, Sarmiento DM, Collen JF, Robben PM, et al Atypical Q fever in US soldiers. Emerg Infect Dis. 2007;13(8):1247–9. 10.3201/eid1308.070218 ; PubMed Central PMCID: PMCPMC2828091. 17953104 PMC2828091

[8] Anderson A, Bijlmer H, Fournier PE, Graves S, Hartzell J, Kersh GJ, et al Diagnosis and management of Q fever—United States, 2013: recommendations from CDC and the Q Fever Working Group. MMWR Recomm Rep. 2013;62(RR-03):1–30. . 23535757

[9] Oyston PC, Davies C. Q fever: the neglected biothreat agent. J Med Microbiol. 2011;60(Pt 1):9–21. 10.1099/jmm.0.024778-0 . 21030501

[10] Fries LF, Waag DM, Williams JC. Safety and immunogenicity in human volunteers of a chloroform-methanol residue vaccine for Q fever. Infect Immun. 1993;61(4):1251–8. ; PubMed Central PMCID: PMCPMC281355. 8454328 10.1128/iai.61.4.1251-1258.1993 PMC281355

[11] Marmion BP. Development of Q-fever vaccines, 1937 to 1967. Med J Aust. 1967;2(24):1074–8. . 4864428 10.5694/j.1326-5377.1967.tb27293.x

[12] Kazar J, Brezina R, Palanova A, Tvrda B, Schramek S. Immunogenicity and reactogenicity of a Q fever chemovaccine in persons professionally exposed to Q fever in Czechoslovakia. Bull World Health Organ. 1982;60(3):389–94. ; PubMed Central PMCID: PMCPMC2536007. 6982774 PMC2536007

[13] Kazar J, Rehacek J. Q fever vaccines: present status and application in man. Zentralbl Bakteriol Mikrobiol Hyg A. 1987;267(1):74–8. . 3324573 10.1016/s0176-6724(87)80190-6

[14] Wilhelmsen CL, Waag DM. Guinea pig abscess/hypersensitivity model for study of adverse vaccination reactions induced by use of Q fever vaccines. Comp Med. 2000;50(4):374–8. . 11020154

[15] Ruble DL, Elliott JJ, Waag DM, Jaax GP. A refined guinea pig model for evaluating delayed-type hypersensitivity reactions caused by Q fever vaccines. Lab Anim Sci. 1994;44(6):608–12. . 7898035

[16] Ascher MS, Williams JC, Berman MA. Dermal granulomatous hypersensitivity in Q fever: comparative studies of the granulomatous potential of whole cells of Coxiella burnetii phase I and subfractions. Infect Immun. 1983;42(3):887–9. ; PubMed Central PMCID: PMC264382. 6642669 10.1128/iai.42.3.887-889.1983 PMC264382

[17] Elliott JJ, Ruble DL, Zaucha GM, Jaax GP, Waag DM. Comparison of Q fever cellular and chloroform-methanol residue vaccines as skin test antigens in the sensitized guinea pig. Acta Virol. 1998;42(3):147–55. . 9842444

[18] Biberstein EL, Crenshaw GL, Behymer DE, Franti CE, Bushnell RB, Riemann HP. Dermal reactions and antibody responses in dairy cows and laboratory animals vaccinated with Coxiella burnetii. Cornell Vet. 1974;64(3):387–406. . 4845729

[19] Ascher MS, Berman MA, Parker D, Turk JL. Experimental model for dermal granulomatous hypersensitivity in Q fever. Infect Immun. 1983;39(1):388–93. ; PubMed Central PMCID: PMCPMC347951. 6822420 10.1128/iai.39.1.388-393.1983 PMC347951

[20] Reeves PM, Paul SR, Sluder AE, Brauns TA, Poznansky MC. Q-vaxcelerate: A distributed development approach for a new Coxiella burnetii vaccine. Hum Vaccin Immunother. 2017;13(12):2977–81. 10.1080/21645515.2017.1371377 ; PubMed Central PMCID: PMCPMC5718828. 28933682 PMC5718828

[21] Schulze LSC, Borchardt S, Ouellet V, Heuwieser W. Effect of a phase I Coxiella bumetii inactivated vaccine on body temperature and milk yield in dairy cows. J Dairy Sci. 2016;99(1):541–50. WOS:000367214700050. 10.3168/jds.2015-9628 26547657

[22] Omsland A, Heinzen RA. Life on the outside: the rescue of Coxiella burnetii from its host cell. Annu Rev Microbiol. 2011;65:111–28. 10.1146/annurev-micro-090110-102927 . 21639786

[23] Kersh GJ, Oliver LD, Self JS, Fitzpatrick KA, Massung RF. Virulence of Pathogenic Coxiella burnetii Strains After Growth in the Absence of Host Cells. Vector-Borne and Zoonotic Diseases. 2011;11(11):1433–8. 10.1089/vbz.2011.0670. ISI:000296923700003. 21867419

[24] Tamrakar SB, Haluska A, Haas CN, Bartrand TA. Dose-response model of Coxiella burnetii (Q fever). Risk Anal. 2011;31(1):120–8. 10.1111/j.1539-6924.2010.01466.x . 20723147

[25] Arricau-Bouvery N, Souriau A, Bodier C, Dufour P, Rousset E, Rodolakis A. Effect of vaccination with phase I and phase II Coxiella burnetii vaccines in pregnant goats. Vaccine. 2005;23(35):4392–402. 10.1016/j.vaccine.2005.04.010. ISI:000230947700004. 16005747

[26] Kersh GJ, Lambourn DM, Raverty SA, Fitzpatrick KA, Self JS, Akmajian AM, et al Coxiella burnetii Infection of Marine Mammals in the Pacific Northwest, 1997–2010. Journal of Wildlife Diseases. 2012;48(1):201–6. ISI:000299136800025. 10.7589/0090-3558-48.1.201 22247392 PMC11288310

[27] Terril LA, Clemons DJ. The Laboratory Guinea Pig. Boca Raton, FL: CCR Press; 1998.

[28] Wahlkvist H, Boman A, Liden C. Dose-response studies of contact allergens using 3 guinea pigs models. Contact Dermatitis. 1999;41(4):198–206. . 10515098 10.1111/j.1600-0536.1999.tb06130.x

[29] Buday T, Gavliakova S, Mokry J, Medvedova I, Kavalcikova-Bogdanova N, Plevkova J. The Guinea Pig Sensitized by House Dust Mite: A Model of Experimental Cough Studies. Adv Exp Med Biol. 2016;905:87–95. 10.1007/5584_2016_217 . 26987338

[30] Uetrecht J. Role of animal models in the study of drug-induced hypersensitivity reactions. AAPS J. 2006;7(4):E914–21. 10.1208/aapsj070489 ; PubMed Central PMCID: PMCPMC2750961. 16594644 PMC2750961

[31] Estrada IC, Gutierrez MC, Esparza J, Quesada-Pascual F, Estrada-Parra S, Possani LD. Use of synthetic peptides corresponding to sequences of Mycobacterium leprae proteins to study delayed-type hypersensitivity response in sensitized guinea pigs. Int J Lepr Other Mycobact Dis. 1992;60(1):18–27. . 1602190

[32] Briand EJ, Ruble GR, Stiteler J, Harris LD, Burge JR, Soranaka ET, et al Comparison of adjuvants with Leishmania antigens in a guinea pig model to induce delayed-type hypersensitivity responses. Lab Anim Sci. 1999;49(5):519–21. . 10551453

[33] Bonhoeffer J, Imoukhuede EB, Aldrovandi G, Bachtiar NS, Chan ES, Chang S, et al Template protocol for clinical trials investigating vaccines—focus on safety elements. Vaccine. 2013;31(47):5602–20. 10.1016/j.vaccine.2013.02.041 ; PubMed Central PMCID: PMCPMC4586124. 23499603 PMC4586124

[34] DMID Interventional Protocol Template. In: Diseases NIoAaI, editor. Version 5.0 ed: National Institute of Health; 2011.

[35] Ascher MS, Berman MA, Ruppanner R. Initial clinical and immunologic evaluation of a new phase I Q fever vaccine and skin test in humans. J Infect Dis. 1983;148(2):214–22. . 6350491 10.1093/infdis/148.2.214

[36] Bell JF, Lackman DB, Meis A, Hadlow WJ. Recurrent Reaction of Site of Q Fever Vaccination in a Sensitized Person. Mil Med. 1964;129:591–5. . 14199980

[37] Lackman DB, Bell EJ, Bell JF, Pickens EG. Intradermal sensitivity testing in man with a purified vaccine for Q fever. Am J Public Health Nations Health. 1962;52:87–93. ; PubMed Central PMCID: PMCPMC1522671. 14461400 10.2105/ajph.52.1.87 PMC1522671

