# Supplementary figures and images for: Standardized guinea pig model for Q fever vaccine reactogenicity
Source: PLoS One. 2018 Oct 12;13(10):e0205882. doi: 10.1371/journal.pone.0205882 (PMC6185858; doi:10.1371/journal.pone.0205882)

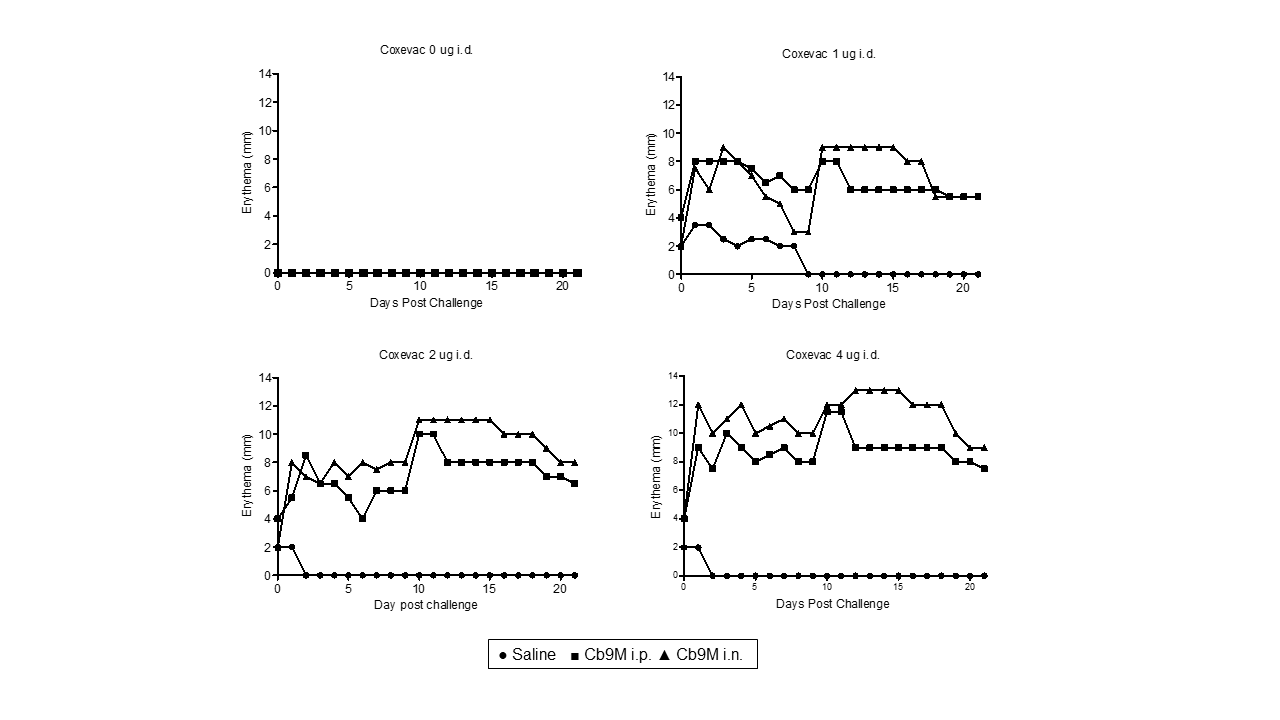

Supplement: S1 Fig — Data are shown as the zone of erythema (mm) at intradermal vaccine injection sites after day 42 p.i. challenge, grouped by challenge dose and route of sensitization with C. burnetii Nine Mile (Cb9M) (i.p., intraperitoneal; i.n., intranasal) or saline controls. Measurements are presented as group mean (n = 2) from day 0 to day 21 post-challenge. (TIF) [file pone.0205882.s001.tif]
